# Bedeutung der Gesetzgebung zur Luftreinhaltung in der Prävention umweltbedingter Erkrankungen

**DOI:** 10.1007/s10405-023-00499-9

**Published:** 2023-03-01

**Authors:** Meltem Kutlar Joss, Nicole Probst-Hensch

**Affiliations:** 1 Dokumentationsstelle Luftverschmutzung und Gesundheit (LUDOK), Departement Epidemiologie und Public Health, Schweizerisches Tropen- und Public Health-Institut, Assoziiertes Institut der Universität Basel, Kreuzstr. 2, 4123 Allschwil, Schweiz; 2 Departement Epidemiologie und Public Health, SAPALDIA Kohorte und Biobank, Schweizerisches Tropen- und Public Health-Institut, Assoziiertes Institut der Universität Basel, Basel, Schweiz

**Keywords:** Luftverschmutzung, Gesundheit, Maßnahmen, Politik, Grenzwerte, Air pollution, Health, Measures, Policy, Air quality standards

## Abstract

Die Luftverschmutzung beispielsweise durch Feinstaub (PM, particulate matter), Stickoxide oder Ozon ist schädlich für die Gesundheit. Bestehende Lungenkrankheiten können sich durch kurzfristig erhöhte Luftbelastung verschlimmern. Langfristige Luftbelastung trägt insbesondere zur Entstehung von kardiorespiratorischen Erkrankungen bei. In Deutschland starben im Jahr 2019 53.000 Menschen vorzeitig aufgrund der Feinstaubbelastung. Die Luftreinhaltung ist eine politische Aufgabe mit großem gesundheitlichem Potenzial. Sie hat in den letzten Jahren wesentlich zur Verbesserung der Luftqualität und damit der Gesundheit beigetragen. In Anbetracht der neuen stringenteren Luftqualitätsleitlinien der Weltgesundheitsorganisation (WHO) sind die Behörden und Politiker nun weltweit mit der Frage der Anpassung der Luftreinhalteziele konfrontiert. In Europa prägt die EU-Direktive die Luftreinhalteziele der Mitgliedstaaten. Die Festlegung der Richtwerte obliegt dem EU-Parlament und dem Rat der EU. Das Nichterreichen der gesetzten Ziele ist mit Strafen verbunden. Deshalb besteht die Gefahr, dass erreichbare und weniger ambitionierte Ziele gesetzt werden. Bereits heute liegen die EU-Richtwerte wesentlich höher als jene in den USA oder der Schweiz. Während „nur“ 11 % der Bevölkerung in der EU einer Belastung über dem EU-Grenzwert für Feinstaub PM10 im Jahr 2020 ausgesetzt waren, sind bei Anwendungen der neuen WHO-Leitlinie 71 % der Bevölkerung übermäßigen und gesundheitsgefährdenden Feinstaubwerten ausgesetzt. Zur wichtigsten und erfolgreichsten Maßnahme der Luftreinhaltung zählt die Reduktion der Luftschadstoffe an der Quelle: die Emissionsbegrenzung. Trotz der Energiekrise dürfen Ziele bezüglich Luftreinhaltung und Klimaschutz nicht aus den Augen verloren werden. Wichtig ist, dass der Gesundheitsschutz nicht dem Einzelnen überlassen werden kann. Gesundheitsfachleute haben in der Beratung empfindlicher Patienten im Umgang mit kurzfristig erhöhter Luftschadstoffbelastung eine wichtige klinische Funktion, aber darüber hinaus ist ihre beratende Rolle in der Politik sehr bedeutsam.

## Abstract (en)

Air pollution, for example from particulate matter, nitrogen oxides or ozone, is harmful to health. Short-term increases in air pollution can lead to exacerbation of existing lung diseases. Long-term air pollution contributes to the development of cardiorespiratory diseases. According to the European Environment Agency, 53,000 people prematurely died in Germany in 2019 due to particulate matter pollution. Air pollution control is a political task with great public health potential. In recent years, it has significantly contributed to improving air quality and thus health. In view of the new more stringent World Health Organization (WHO) air quality guidelines, the authorities and policy makers worldwide are now confronted with the question of adjusting air quality targets and setting standards. In Europe, the EU Directive on air quality standards is passed by the EU Parliament and the Council of the EU and is binding for air quality targets of member states. Member states can be brought to court for failure to achieve the targets. Therefore, there is a risk that achievable and less ambitious air pollution targets will be set. Even now, the EU guideline values are significantly higher than those in the USA or Switzerland. While “only” 11% of the EU population were exposed to levels above the current EU limit for PM10 in 2020, 71% of the population were exposed to hazardous levels of PM10 following the new recommendation by the WHO. Among the most important and successful air pollution control measures is the reduction of air pollutants at the source: emission control. Despite the energy crisis goals regarding air pollution control and climate protection must not be ignored. Importantly, health protection cannot be left to individuals. Health professionals have an important clinical role in advising sensitive patients on how to deal with short-term elevated levels of air pollutants but beyond that their advisory role in policy is very significant.

## Luftverschmutzung und Gesundheitsfolgen

Luftverschmutzung stammt aus einer Vielzahl von natürlichen und anthropogenen (vom Menschen verursachten) Emissionsquellen. Die wichtigsten Quellen der anthropogenen Luftverschmutzung in Deutschland umfassen industrielle Prozesse (Feinstaub PM [„particulate matter“], NO_x_ [Stickoxide], flüchtige organische Kohlenwasserstoffe VOC [„volatile organic compounds“], SO_2_ [Schwefeldioxid]), die Energiegewinnung für Industrie und Haushalte (PM, NO_x_, SO_2_, Ruß), den motorisierten Verkehr (NO_x_, PM) und die Landwirtschaft (NH_3_ [Ammoniak] ein Vorläufer von Feinstaub, PM) [1].

Luftverschmutzung ist die Verunreinigung der von uns eingeatmeten Innen- und Außenluft durch chemische, physikalische oder biologische Komponenten mit potenziell bedrohlichen Folgen für die Gesundheit des Menschen und des Ökosystems. Zu den Schadstoffen, bei denen es die deutlichsten Belege für gesundheitliche Bedenken gibt, zählen Feinstaub (PM), Ozon (O_3_), Stickstoffdioxid (NO_2_) und Schwefeldioxid (SO_2_) sowie Kohlenmonoxid (CO).

Die durch Feinstaub bedingten Gesundheitsrisiken sind für die Gesundheit des Einzelnen und für die öffentliche Gesundheit von besonderer Bedeutung. PM2.5 und PM10 (Feinstaub, der nicht größer als 2,5 bzw. 10 μm im Durchmesser misst) können bis tief in die Lunge vordringen, ultrafeine Partikel können in den Blutkreislauf gelangen. Feinstaub und Außenluftverschmutzung wurden auch als krebserregend eingestuft [2]. Es gibt mittlerweile viele biologische Wirkungsmechanismen, welche die beobachteten Gesundheitseffekte in den Atemwegen, aber auch im Herz-Kreislauf-System und anderen Organen erklären [3].

Feinstaub kann je nach Größe sehr tief in die Lunge vordringen

**Figure 1:**
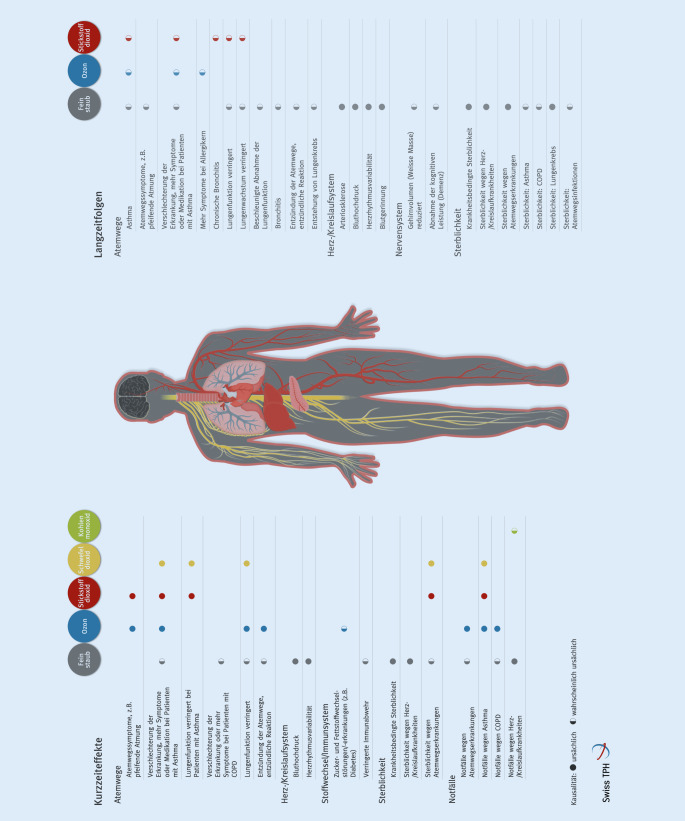

Die lufthygienische Dokumentationsstelle am Swiss TPH (Schweizerisches Tropen- und Public Health-Institut) hat die mit mittlerer bis hoher Wahrscheinlichkeit kausalen Gesundheitsfolgen der Luftschadstoffe in einer Infografik zusammengetragen (Abb. 1; [4]; https://www.swisstph.ch/de/projects/ludok/healtheffects/). Es gibt mittlerweile kein Organsystem, das nicht von den schädlichen Auswirkungen der Luftverschmutzung betroffen wäre.

Für die pneumologische Praxis in Westeuropa sind v. a. folgende Effekte relevant: Einerseits kommt es zu Exazerbationen bestehender Lungenerkrankungen wie COPD (chronisch obstruktive Lungenerkrankung) oder Asthma bei kurzfristig erhöhten Belastungen mit den Schadstoffen Feinstaub, Ozon, NO_2_ und SO_2_. Solche erhöhten Belastungen kommen z. B. durch Schwankungen der Wetterlage zustande. So ist an Tagen mit erhöhter Belastung mit einer Zunahme von Arztkonsultationen, Notfällen, Spitaleintritten und Todesfällen zu rechnen. Pro 10 μg/m^3^ Belastungsanstieg von PM2.5, NO_2_ oder Ozon ist mit einer Zunahme der Asthmaexazerbationen um 1–6 % zu rechnen [5–7]. Auch kurzfristige Aufenthalte von wenigen Stunden in stark belasteten Situationen, wie z. B. einer hohen Straßenverkehrsbelastung, können zu nachweisbaren Verringerungen der Lungenfunktion führen [8]. Schwerwiegender aus Public-Health-Perspektive ist der Beitrag der langfristigen Luftverschmutzung zur Entwicklung chronischer Lungenkrankheiten wie Asthma und COPD und einer erhöhten Sterblichkeit u. a. an Atemwegserkrankungen [9].

## Rolle der medizinischen Versorgung

Je nach individueller Empfindlichkeit, Vorerkrankung oder Prädisposition reagieren Personen unterschiedlich stark auf Luftverschmutzung. Wo einzelne nur milde Symptome wie ein Halskratzen bei erhöhten Ozonwerten spüren, können andere bereits von einem Asthmaanfall betroffen sein. Wichtig ist, die Patienten zu möglichen Folgen erhöhter Luftbelastung aufzuklären und beispielsweise Notfallmaßnahmen wie das Mitführen eines Inhalators bei Asthma zu empfehlen. Außerdem sollten Patienten die tägliche Luftqualität im Auge behalten und bei Bedarf ihre Aktivitäten anpassen. So sollten z. B. sportliche Aktivitäten an ozonreichen Sommertagen eher in die Morgenstunden verlegt werden (s. auch [10]). Für das Monitoring der Luftqualität eignen sich Luftqualitätsindizes (z. B. https://www.umweltbundesamt.de/daten/luft/luftdaten).

## Rolle der Politik

Die wichtigste und wirksamste Maßnahme zur Bekämpfung der Luftschadstoff-bedingten Krankheitslast ist die nachhaltige Verbesserung der Luftqualität durch Verminderung der Emissionen und Festlegung von bindenden Luftqualitätsgrenzwerten bezüglich der Immissionen. Die USA und Europa haben zudem gezeigt, dass Umwelt- und damit Gesundheitsschutz mit wirtschaftlichem Wachstum vereinbar ist [11]. Das individuelle Risiko, durch Luftverschmutzung zu erkranken, ist zwar klein gegenüber anderen Faktoren wie beispielsweise dem Lebensstil (Rauchen, Bewegung, Ernährung). Da jedoch alle Personen von jung bis alt ständig und mehr oder weniger unausweichlich der Belastung mit Luftschadstoffen ausgesetzt sind, summieren sich diese Risiken zu einer großen Gesundheitslast auf Bevölkerungsebene. Hinzu kommen die Risiken von akuten Exazerbationen bei bereits Erkrankten.

Weltweit ist die Luftverschmutzung der wichtigste Umweltrisikofaktor

Weltweit ist die Luftverschmutzung der wichtigste Umweltrisikofaktor, verantwortlich für über 6,5 Mio. vorzeitige Todesfälle [12]. Die europäische Umweltagentur beziffert die Zahl der vorzeitigen Todesfälle aufgrund der Feinstaub, NO_2_- und Ozonbelastung in der EU für das Jahr 2021 auf 307.000, 40.400 respektive 16.800 vorzeitige Todesfälle [13].

Die Weltgesundheitsorganisation (WHO) definiert seit 1987, basierend auf dem aktuellen Stand des Wissens, Luftqualitätsleitlinien („air quality guidelines“ [AQG]) zum Schutz der Gesundheit; zuletzt 2021 [14]. Basierend auf der aktuellen Evidenz aus Hunderten von epidemiologischen Studien haben systematische Übersichtsarbeiten zu Sterblichkeit, Notfällen wegen Asthma oder Herzinfarkten gezeigt, dass die negativen Gesundheitsfolgen selbst bei sehr niedrigen Belastungen noch bestehen. Die WHO fordert daher für den Gesundheitsschutz, die langfristige Schadstoffbelastung mit Feinstaub (Partikel mit einem Durchmesser 2,5 µm oder weniger PM2.5) auf ein Jahresmittel von unter 5 μg/m^3^ zu reduzieren, jene mit NO_2_ auf unter 10 μg/m^3^ und jene mit Ozon auf ein Mittel von unter 60 μg/m^3^ in den Sommermonaten (Tab. 1) zu begrenzen. Diese Werte sollen eingehalten werden, um die Bevölkerung vor schwerwiegenden Gesundheitsschäden durch Luftverschmutzung zu schützen. Dabei sollen die Luftqualitätsleitlinien als Vorlage für die Formulierung nationaler Grenzwerte dienen. Die WHO-Leitlinien sind selber nicht bindend.

**Table 1:** 

Schadstoff	Mittelungszeit	AQG-Leitwert 2005	AQG-Leitwert 2021	EU-Richtlinie 2008	Vorgeschlagene Werte der EU Stand 26.10.2022
PM2.5 (µg/m^3^)	Jahr	10	5	25	10
	24 h^a^	25	15	–	25
PM10 (µg/m^3^)	Jahr	20	15	40	20
	24 h^a^	50	45	50	45
Ozon (µg/m^3^)	Sommersaison^b^	–	60	–	–
	8‑h-Maximum^a^	100	100	120^c^	120^c^
NO_2_ (µg/m^3^)	Jahr	40	10	40	20
	24 h	–	25^a^	200 (1-h-Mittelwert)	50
SO_2_ (µg/m^3^)	Jahr	20	–	20 (kritischer Wert zum Schutz der Vegetation)	20
	24 h	–	40^a^	125	50
CO (mg/m^3^)	24 h	–	4^a^	10 (8-h-Mittelwert pro Tag)	4

*PM* „particulate matter“, *NO*_*2*_ Stickstoffdioxid, *SO*_*2*_ Schwefeldioxid, *CO* Kohlenmonoxid

^a^99. Perzentil (d. h. 3 Überschreitungstage pro Jahr sind zulässig)

^b^Durchschnitt der maximalen täglichen 8‑h-Mittelwerte der Ozon-Konzentration in den sechs aufeinanderfolgenden Monaten mit der höchsten Ozon-Konzentration im Sechsmonatsdurchschnitt

^c^Zielwert, kein Richtwert

### Festlegung von Luftqualitätsrichtwerten in der EU

In der EU ist das maßgebliche Instrument die Ambient Air Quality Directive (Richtlinie über Luftqualität und saubere Luft), die im Jahr 2008 Richtwerte für die wichtigsten Schadstoffe formulierte [16]. Die festgelegten Richtwerte liegen deutlich höher als die von der WHO formulierten Leitlinien. Insbesondere der Langzeitwert für Feinstaub PM10 (40 μg/m^3^) lag bei seiner Festlegung bereits über der damals gültigen WHO-Leitlinie von 20 μg/m^3^ (vgl. Tab. 1). Auch die von der Europäischen Kommission neu anvisierten Werte der EU-Direktive, die am 26.10.2022 veröffentlicht wurden, liegen noch über den aktuellen Werten der WHO [17]. Neben den Richtwerten legt die Direktive fest, wie und wo die Schadstoffe zu messen bzw. zu beurteilen sind, dass bei Überschreitung der Werte entsprechende Luftreinhaltepläne, lokal, aber auch grenzüberschreitend mit Maßnahmen zur Reduktion der Belastung formuliert werden müssen, sowie Sanktionen bei längerer Nichterreichung der Richtwerte.

Die Inhalte der Direktive sind mit einer festgelegten Frist in nationales Recht umzusetzen, in Deutschland beispielsweise in Form der von der Bundesregierung verabschiedeten Verordnung über Luftqualitätsgrenzwerte und Emissionshöchstmengen (39. BImSchV). Dabei dürfen die nationalen Grenzwerte durchaus strenger sein, aber nicht höher als die von der EU-Direktive gesetzten Richtwerte.

**Figure 2:**
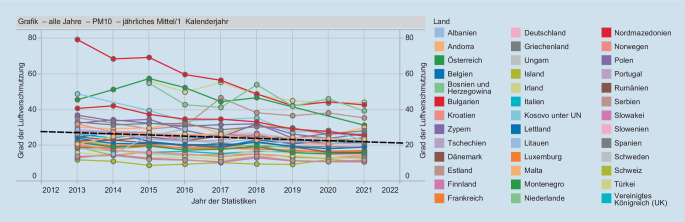

Der Vorteil dieser in der ganzen EU gültigen Grenzwerte ist, dass im einheitlichen Wirtschaftsraum die gleichen Umweltmindeststandards gelten und einheitliche Lebensbedingungen für die Bürger geschaffen werden sollen. Ein Abwandern umweltbelastender Industriebetriebe in EU-Länder mit laxerer Gesetzgebung wird damit verhindert. In der ganzen EU ist in den letzten 10 Jahren ein Rückgang der Feinstaubimmissionen zu verzeichnen (Abb. 2), wobei manche Länder größere Fortschritte aufweisen konnten als andere.

Die EU-weit gültigen Richtwerte werden durch das EU-Parlament sowie den Rat der EU verabschiedet. Also sowohl die Mehrheit des Parlaments als auch die Ländervertretung im Rat der EU müssen der Höhe der Richtwerte zustimmen.

Dies kann auch als Nachteil ausgelegt werden. Da empfindliche Strafen bei Nichteinhaltung der Grenzwerte drohen, kann es sein, dass Grenzwerte so hoch angesetzt werden, dass sie in nützlicher Frist erreichbar sind. In der aktuellen EU-Direktive besteht darüber hinaus kein Anreiz für die weitere Verbesserung der Luftqualität unterhalb der Grenzwerte. Sind die Grenzwerte eingehalten, werden Maßnahmen wie Luftreinhaltepläne aufgehoben. Es besteht kein gesetzlicher Auftrag und Anreiz, die Luftqualität weiter zu verbessern.

### Festlegung von Luftqualitätsgrenzwerten in den USA und der Schweiz

In Ländern wie den USA oder der Schweiz kommt dem Parlament keine Rolle bei der Festlegung der Höhe der Grenzwerte zu. In den USA werden die WHO-Leitwerte nicht als wissenschaftliche Grundlage herangezogen. Es werden für jeden Schadstoff etwa alle 5 Jahre sog. Integrated Science Assessments verfasst, welche die wissenschaftliche Evidenz und die Kausalität beobachteter Zusammenhänge zwischen Schadstoffen und gesundheitlichen Endpunkten beurteilen. Hierzu werden epidemiologische und experimentelle Studien sowie Tier- und Zellstudien herangezogen. Die Umweltschutzbehörde schlägt dann, basierend auf den Ergebnissen und den Policy Optionen, die sich für Maßnahmen und Vollzug anbieten, Grenzwerte vor, die nach einer intensiven Phase der öffentlichen Beratschlagung durch die Umweltbehörde festgelegt werden.

**Figure 3:**
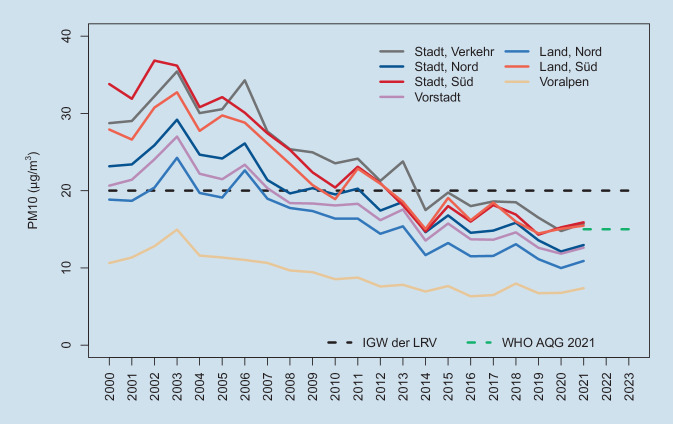

In der Schweiz werden die WHO-Leitwerte als wissenschaftliche Grundlage herangezogen, und ein Expertengremium (Eidgenössische Kommission für Lufthygiene) schlägt Grenzwerte dem Exekutivorgan (Bundesrat) vor, die in einer Verordnung (Luftreinhalteverordnung [19]) nach öffentlicher Anhörung (Vernehmlassung) vom Bundesrat beschlossen werden. Im Vergleich zur EU (Abb. 2) nahm die Luftbelastung mit Feinstaub PM10 in der Schweiz stärker ab (Abb. 3).

## Bedeutung der Grenzwerte für den Gesundheitsschutz

**Figure 4:**
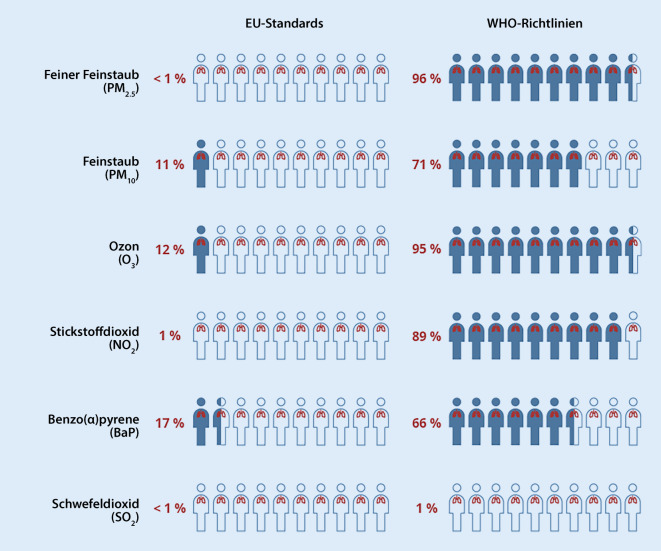

Da die neuen WHO-Luftqualitätsleitlinien gesundheitliche Schäden auch unterhalb der EU-Standards aufzeigen, geht mit einer unveränderten aktuellen EU-Direktive ein enormes Gesundheitspotenzial verloren. Während die tatsächliche Belastung der europäischen Bevölkerung inzwischen im Vergleich zu den gesetzlichen Vorgaben der EU relativ gut aussieht und beispielsweise „nur“ 11 % der Bevölkerung einer Belastung oberhalb des EU-Grenzwertes für Feinstaub PM10 im Jahr 2020 ausgesetzt waren, zeigt sich in Bezug auf die WHO-Leitlinie, dass 71 % der EU-Bevölkerung übermäßigen und gesundheitsgefährdenden Feinstaubwerten ausgesetzt waren (Abb. 4).

### Maßnahmen und das Recht auf saubere Luft

**Figure 5:**
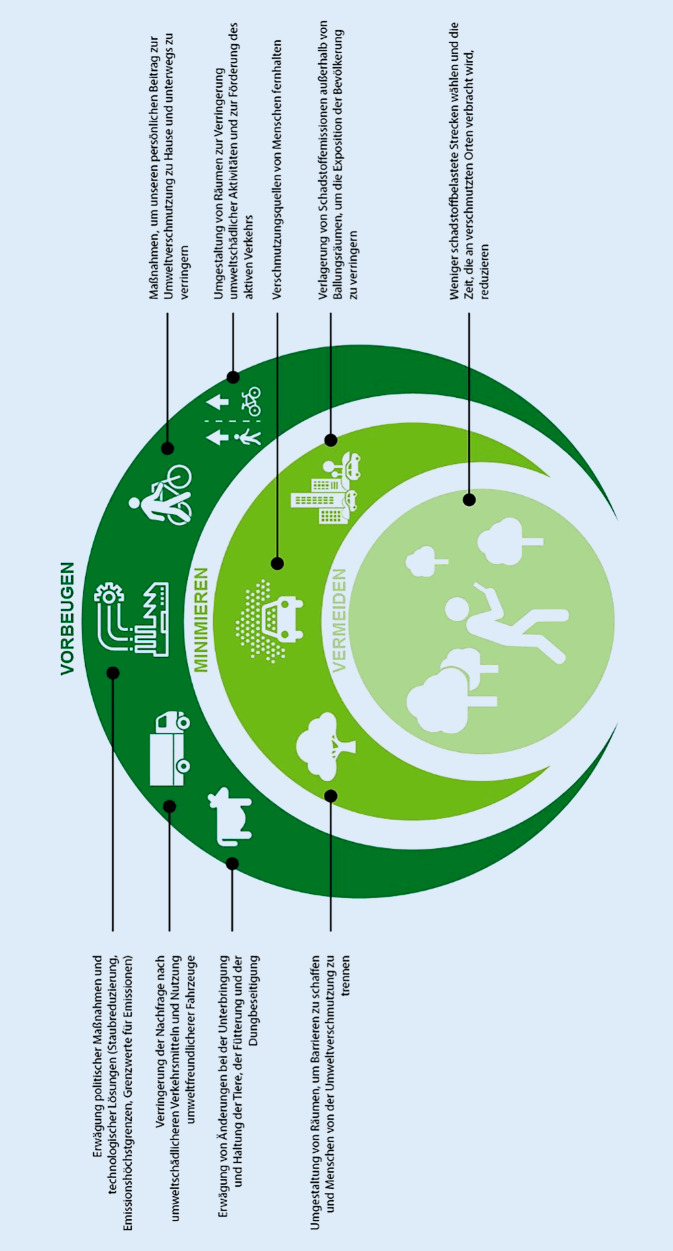

Es gibt nicht eine Maßnahme, die zur Verbesserung der Luftqualität führt. Vielmehr ist es ein Mosaik von Bausteinen. Public Health England, eine öffentlich finanzierte Institution, die inzwischen ersetzt wurde durch UK Health Security Agency and Office for Health Improvement and Disparities, hat die erfolgversprechendsten Maßnahmen und ihre Evidenz in einem Rapid Assessment beurteilt und kam zum Schluss, dass die Verringerung der Emissionen an der Quelle die beste Strategie sei. Maßnahmen zur Verringerung der Immissionen und schließlich die Vermeidung von Belastung durch das Individuum sind diesem Prinzip untergeordnete Maßnahmen ([22]; Abb. 5).

In Deutschland sind die Grenzwerte einklagbar

Die Deutsche Umwelthilfe (DUH) hat seit 2005 durch Musterklagen in verschiedenen deutschen Städten das individuell einklagbare Recht auf saubere Luft erstritten und 2007 durch das Bundesverwaltungsgericht und 2008 durch den Europäischen Gerichtshof bestätigen lassen. Werden die seit 2005 für Feinstaub (PM10) und seit 2010 für Stickstoffdioxid geltenden Grenzwerte für Luftschadstoffe überschritten, können betroffene Bürgerinnen und Bürger in Deutschland ihr „Recht auf saubere Luft“ und damit wirksame Maßnahmen zur Verringerung der Luftschadstoffe einklagen [23].

Durch die Klagen standen die Behörden unter Zugzwang, sodass Einzelmaßnahmen zur Einhaltung von Grenzwerten im Vordergrund standen. Neben dem Einrichten von Dieselfahrverboten in Städten wurden unter anderem sehr fragwürdige Maßnahmen ergriffen, damit an einem Messstandort ein Grenzwert eingehalten werden konnte. In Hamburg wurde beispielsweise die Verkehrsführung geändert, damit weniger Verkehr am Messort vorbeiführt. Dabei wurde der Verkehr weg von der Hauptstraße in andere Gebiete verlagert. Der Grenzwert am Messort wurde daraufhin zwar eingehalten, die veränderte Verkehrsführung führte aber zu einer zusätzlichen Belastung, die eine Gegend ohne Messstation traf. Insgesamt führte dies also nicht zu einem Gesundheitsgewinn, sondern im schlimmsten Fall sogar zu einer zusätzlichen Belastung (vgl. [24]). An der verkehrsbelasteten Messstelle Stuttgart Neckartor wird die Luft seit Ende 2018 mit 23 Luftfiltersäulen von Feinstaub und NO_2_ mit Kosten von insgesamt 1,5 Mio. € gereinigt. Die Feinstaubbelastung sank um durchschnittlich mehr als 10 % und jene mit NO_2_ um 9 % [25]. Neben der Unsinnigkeit solcher Messwertkosmetik ist der Energieverbrauch für den Betrieb bei bestehender Klimakrise und Energiemangellage ein zusätzlicher kontraproduktiver Faktor.

In der Schweiz sind die Grenzwerte nicht einklagbar. Das Umweltschutzgesetz der Schweiz fordert die Festlegung von Immissionsgrenzwerten nach dem Stand der Wissenschaft oder der Erfahrung so, dass Menschen, Tiere und Pflanzen, ihre Lebensgemeinschaften und Lebensräume nicht gefährdet werden und dabei auch Wirkungen auf Personengruppen mit erhöhter Empfindlichkeit (z. B. Kinder, Kranke etc.) berücksichtigt werden [26]. Die Behörden sind verpflichtet, nach dem Vorsorgeprinzip auch dann noch Maßnahmen zur Reduktion der Belastung zu ergreifen, wenn die Grenzwerte erreicht und die Maßnahme verhältnismäßig ist [27]. Die Schweiz konnte in den letzten 30 Jahren enorme Erfolge in der Luftreinhaltung vorweisen (vgl. [28] sowie Abb. 3).

## Schlussfolgerung und Ausblick

Angesichts der hohen Gesundheitslast und -kosten, die der Luftverschmutzung anzulasten sind, sollte sie energischer angegangen werden. Gemäß Europäischer Umweltagentur starben im Jahr 2019 etwa 53.000 Menschen vorzeitig aufgrund der Feinstaubbelastung in Deutschland [29]. Diese Zahl der vorzeitigen Todesfälle entspricht im Prinzip der Gesundheitslast durch die Corona-Pandemie, die in den letzten 3 Jahren über 150.000 Todesfälle in Zusammenhang mit dem Corona-Virus (COVID-19) verzeichnete [30]. Die Lockdowns während der Corona-Pandemie haben vielerorts gezeigt, dass die Luftbelastung mit bestehenden Mitteln merklich verbessert werden kann [31], und in den verkehrsberuhigten Städten stieg die Lebensqualität aufgrund von weniger Lärm- und Luftbelastung, sodass manche Städte, z. B. Mailand, diesen Effekt weitertragen wollen, indem sie mit Pop-up-Rad- und Gehwegen den emissionsarmen Individualverkehr fördern [32].

Die durch den Ukrainekrieg verursachte Energiekrise stellt den Luft- und Klimaschutz vor die Herausforderung, Lösungen nicht zulasten derselbigen zu verfolgen. Der Anstieg der Verkaufszahlen von Holzöfen, das Aussetzen von Emissionsgrenzwerten oder maximalen Laufzeiten bei mobilen Kraftwerken, Notstromgeneratoren oder Gaskraftwerken, die Förderung des Kohleabbaus trotz Ausstiegsversprechen sind einige Beispiele dafür, welche die Erfolge der Luftreinhaltepolitik gefährden.

Saubere Luft ist ein wichtiger Faktor für die Gesundheit. In diesem Sinne kommt den Ärztinnen und Ärzten eine wichtige Aufklärungsrolle auf individueller, aber auch struktureller Ebene zu. Sie können ihre Patientinnen und Patienten beraten und beispielsweise zu körperlicher Aktivität an weniger belasteten Straßen raten. Die Meinung der Fachleute in Gesundheitsfragen kann ebenfalls politische Entscheidungsprozesse beeinflussen und Maßnahmen zur Emissionsminderung und Belastungsreduktion stützen. Das Setzen von ambitionierten Luftqualitätszielen ist dabei ein wichtiges Instrument.

## Fazit für die Praxis

- Saubere Luft ist ein wichtiger Faktor für die Gesundheit.
- Ein kurzfristiger Anstieg der Luftbelastung kann zu einer Zunahme von Exazerbationen und Komplikationen bei Patientinnen und Patienten mit chronischen Lungenerkrankungen führen.
- Die langfristige Luftbelastung erhöht das Risiko, chronische Lungenkrankheiten zu entwickeln.
- Zum Schutz der Patient:innen und Bevölkerung muss die Luftbelastung reduziert werden.
- Luftqualitätsgrenzwerte mit entsprechenden Maßnahmen sind ein wichtiges Instrument der Prävention.
- Die Reduktion der Emissionen an der Quelle ist die kostengünstigste und effektivste Maßnahme.
- Patieninnen und Patienten können die Folgen kurzfristig erhöhter Luftbelastung durch angepasste Medikation und Verhalten beeinflussen.
- Ärztinnen und Ärzte haben eine wichtige Aufklärungsrolle auf individueller Ebene, können aber auf politischer Ebene politische Entscheidungsprozesse in Richtung gesunde Luftqualität unterstützen.
